# The role of parental involvement in academic and sports achievement

**DOI:** 10.1016/j.heliyon.2024.e24290

**Published:** 2024-01-08

**Authors:** Klára Kovács, Ádám József Oláh, Gabriella Pusztai

**Affiliations:** a MTA-DE-Parent-Teacher Cooperation Research Group, Institute of Educational Studies and Cultural Management, Faculty of Humanities, University of Debrecen, Egyetem sqr. 1, 4032, Debrecen, Hungary; b MTA-DE-Parent-Teacher Cooperation Research Group, Institute of Sports Science, University of Debrecen, Egyetem sqr. 1, 4032 Debrecen, Hungary

**Keywords:** Parental involvement, Academic achievement, Sports performance, Engagement in sports, Primary school pupils

## Abstract

According to previous research results and our systematic review on this topic (Kovács et al., 2022), the positive impact of parental involvement on academic achievement was revealed. However, it is unclear how parental involvement in sports contributes to sports performance and academic achievement. In this study, our main questions are about the differences in academic achievement, in participation in sports activity, and in sports results among pupils of parents involved or not involved in sports and education. To answer these questions, a questionnaire survey was conducted among 7th and 8th-grade students learning in three primary schools in a Hungarian city (N = 121). Based on the scale of parental involvement, three groups were differentiated: 1) children of parents not involved in either education or sports (N = 33), 2) children of parents involved in education only (N = 38), and 3) children of parents involved in both education and sports (N = 47). In order to examine this data, cluster and factor analysis, a Chi-square test and ANOVA, and linear regression were used. Our results showed, children of parents involved in both sports and education are overrepresented among students who received awards because of their sports performance (59.6%), among members of sports talent management programs (29.8%), and among those who achieved first place in national sports competitions (47.8%). They also seem to be the ones most intent on staying engaged, in the future, in regular and competitive sport activities (57.4% and 55.3%). On the contrary, the highest percentage of children of parents involved in education belongs to those who were given awards because of excellent academic achievement (71.1%). As far as personality traits are concerned, obsessive passion (OP) is the most characteristic of pupils with non-involved parents (M = 0.37, SD = 0.95) and least characteristic of children with parents involved in education (M = −0.17, SD = 0.94). Belonging to both groups with involved parents can be considered a negative predictor of OP (βt = −0.259* [−1.019, −0.078], βt + s = −0.237* [−0.930, −0.030]). In conclusion, it can be detected that there exists a positive interrelation between parental involvement in sports and education, and the academic and sports performance of their children.

## 1 Introduction

Previous research has focused on the forms of parental involvement and their impact, while also examining the positive effects of sports on health and academic achievement. Parents' capital, their support of their children, and their involvement in their children’s lives increases their child’s ambition to perform better at school and reduces the likelihood of dropout [[1], [2], [3], [4], [5], [6], [7]]. It was also found that students who participate in extracurricular activities have better academic achievement than those who do not, and have less disadvantage compared to their peers. However, more information is needed on parental involvement in extracurricular sports activities [[8], [9], [10]]. Our research aims to explore the extent of their involvement in their child’s sporting activities and its impact on different dimensions of student sports and academic achievement.

The relevance of our research is supported by the fact that several studies have already examined the form and extent of parental involvement in, and the impact of parental’ social background on, children’s academic achievement [11,2,6,7,12]. Studies have also been carried out regarding the involvement of parents in and the impact of targeted programmes on students' physical activity, nutrition, and body mass index [13]. However, in our previous systematic review, we did not find any research that examined the impact of parental involvement in school sports on students' academic achievement [11]. Thus, in our current study, we investigate the differences, in social and sporting background, academic and sporting achievement, passion for sport and vision for the future, between children of parents who are and are not involved in sports and education.

### 1.1 Parenting, parental involvement and academic achievement

According to Epstein's theory of parenting, parental involvement is composed of six dimensions [14], of which parenting itself is the main concern of our research. This includes activities that create a home environment for the child, which is necessary for optimal physical, emotional and intellectual development, e.g. reading stories, playing together, going on trips, playing music and playing sports together. These contribute to the development of an intimate, loving parent-child relationship. Baumrind [15] distinguished between three parenting styles (PS) [11]: authoritative (controlling, demanding, communicative, and nurturant) [1], non-authoritative (non-nurturant), and [2] permissive (non-controlling). According to the author's research, adolescent children of authoritative parents were more likely to be mature, successful, communicative, and social than children of non-authoritative parents, while preschool children of permissive parents were more likely to be characterised by low self-confidence, self-control, and competence when moving into adolescence. A new concept in parenting literature stemming from positive psychology is strength-based parenting (SBP), which differs from authoritative parenting in that, while the latter develops warm, nurturing, and responsive parent-child relationships, strength-based parenting motivates children to become aware of their personal strengths, and seek opportunities to use those strengths in real-life situations. Its main characteristic is that the parent is aware of the child's personality, abilities and talents, and encourages their use [16]. The concept is particularly relevant to our research because it has been transferred from sports research to educational psychology [17]. We also assume that if the parent knows that the child's strengths lie in sports and/or learning, and supports their child in these areas, encouraging him or her to use them, then the child will be more successful. Waters and Sun [18] introduce the concept of strengths deployment, which shows the extent to which individuals deploy strengths within a given role. They cite the example of when a sporty parent (as a strength) coaches their child's sporting team.

The impact of a parenting style on student achievement has been studied in several researches as well. Baumrind [15] found higher academic achievement among children of authoritative parents. A scoping review concluded that articles addressing parenting style and achievement focus on five topics [11]: parental control is dependent on style and leads to achievement [1], gender of parents influences style and achievement [2], parents' educational attainment influences style and achievement [3], parents and their children perceive parenting style differently, and [4] style and achievement are influenced by ethnicity and diversity [19]. Strength-based parenting was found to be indirectly related to higher school engagement [17], negatively to stress levels of elementary school children and positively to strength-based coping mechanisms, thus enhancing personal strengths that help children overcome stressful obstacles [20]. Results from a survey of Australian secondary school students found that SBP has a direct positive effect on adolescent well-being (engagement and perseverance) while having an indirect positive effect on academic achievement (grades) through perseverance [16].

According to Nechyba et al. [21], parental support is the primary determinant of children's school performance. Parental support is determined by the personality of the child's family members, including that of the parents. These factors can lead to three possible outcomes, which can occur simultaneously or separately. Family and parent involvement in the child's life changes the child's personality (through parenting and inherited traits), determining the child's academic achievement. The family may be involved in the development of the community, which can develop the quality of the school and, thus by proxy, the personality of the child. The involvement of parents in daily life and activities at school also directly affects the quality of the educational institution, thereby enriching the child's personality whilst increasing chances of his or her success [21]. This shows that family, school, and community form a strong interdependent system around the child. School-family-community cooperation, i.e. parental involvement (in these areas), comprises six dimensions: 1. Parenting: tasks, which include providing housing, safety, and opportunities (in our research, this is what parenting means); 2. Communication: effective information transfer between the actors involved (parent-child, parent-school); 3. Volunteering: the parent's active involvement in school work; 4. Learning at home: learning where the parent provides quality help with homework and preparation for school work; 5. Decision-making: the parent's membership and activity in school boards; 6. Collaboration: cooperation with the community linking with the wider community institutions surrounding the school [22].

However, the impact of parental involvement on academic achievement varies along with parental style. Its effect on academic achievement is higher among children of authoritative parents than those of non-authoritative parents. This may be because authoritative parents more easily interact with adolescents when they go to school, choose a specialisation, and are better able to understand their problems, thus helping young adults to make good decisions. In a study, in Serbia, the authors also found that mothers with an authoritative parenting style were more involved in school activities, and their children were more successful. In contrast, fathers with an authoritative parenting style tended to lack time to participate in school programs. Consequently, the authors conclude that schools need to provide parents with relevant information about the many ways parenting style affects children’s academic achievement; for this briefing to take place, the development of an appropriate partnership between school and family is required [23].

Parental involvement is influenced by several factors categorized into groups. One major group, subjective factors, includes differences in parents' views of the child's future and the role of the school in their development. The other main group of factors includes socio-cultural and ethnic background, economic disadvantages, low educational degree, lack of positive school experience, lack of information, uncertainty in the educational process, the instability of relationships and daily routines within the family, and the lack of recreational cultural consumption [24].

### 1.2 Parental involvement and sports performance

The impact of parental involvement on sports performance is ambivalent. According to Talha et al. [25], parental encouragement and support, positive responses to problems, belief in children's abilities and motivation play an essential role in their sporting careers and performance. However, rather than highlighting the active involvement of parents in their child’s sporting activities, other research findings point to the role of parental attitudes (e.g. praise or understanding) in children's acceptance of their parents' (sporting) values. Empathic, understanding attitudes are associated with a more positive parent-child relationship, whereas too much pressure on children regarding sport may make them unwilling to share his/her experiences, difficulties and/or problems with the parents. The latter thus worsens the parent-child relationship [26]. A Danish longitudinal study also clearly demonstrated the positive role of parental involvement in children's sport participation. However, the association was different when children of disadvantaged and advantaged parents were examined separately. For disadvantaged children, parental involvement had a positive effect on children's involvement in sports, whereas the opposite was found for parents from better social backgrounds [27]. Concerning parental pressure, it was found that the pressure from parents on their children is a significant predictor of burnout syndrome, particularly among Turkish athletes [28]. However, the association of SBP with sports performance tends to present a positive picture. In the sports literature, the strength-based approach (SBA) refers to an environmental factor that contributes to and enhances mental toughness. It refers to the experiences and environments that provide opportunities for a child to recognize and use their strengths while developing mental toughness [17]. SBA in sports (also) focuses on strengths rather than weaknesses, even when the result is negative [29].

Our research examines the dualistic model of passion for sport [30] concerning different forms of parental involvement in sport performance. Passion in sport is essential for an athlete to function optimally. It is strongly connected to motivation, resting on the fact that people engage in certain activities in the hope of satisfying the need for autonomy (a desire to feel a sense of personal initiative), competence (a desire to interact effectively with the surrounding environment), and belonging (a desire to feel connected to significant others), as is laid out in the self-determination theory of Deci and Ryan [31]. An athlete with passion often has outstanding performance, and this can have a positive impact on psychological well-being. However, some forms of passion, where the outstanding performance associated with passion has a severe psychological cost, can reduce psychological well-being. Accordingly, the dual model of passion distinguishes between two types: 1) harmonious passion (HP) for an activity is when the activity plays a significant (not all consuming) role in the individual's life and identity. The person has full control of his or her passion for the activity, which flows and matches with other aspects of his or her life. In HP, the individual can accept and engage in the activity without developing an addiction for it and without participation in it getting out of control. In contrast, obsessive passion (OP) is an uncontrollable urge on the part of the individual to engage in an activity that he or she likes and finds enjoyable, so much so that the commitment to the activity is uncontrolled. Because everything revolves around passionate activity, OP is associated with strong persistence, even if this engagement is already detrimental to other areas of one's life, goals and activities [30,32].

Based on the literature presented above, our research aims to explore the sociocultural background and the role of parental involvement in different dimensions of students' sports and academic achievement. We formulated the following research questions: what groups of students can be identified regarding parental involvement? What differences in parental involvement can be found pertaining to the socio-demographic and athletic backgrounds of pupils? What are the differences in academic and athletic performance between groups of pupils differentiated by parental involvement? What role does SBP play in these differences? Based on previous research and theoretical models, the following hypotheses are formulated:

H1 Children of parents who are involved in studies and sports have a better social background (more of them come from better financial backgrounds, are children of highly qualified parents, and live in urban areas), while children of parents who are not involved tend to have a lower social status.

H2 Children of parents who are involved in studies are the most successful, while children of parents who are not involved are the least successful in academic achievement.

H3 Children of parents involved in sport are more successful in sport, more consciously plan their future in sport and are more characterised by HP, while OP is less typical.

## 2 Methodology

### 2.1 Data and procedure

In our research, we originally sampled six primary schools in a large city in Hungary, using stratified group sampling. We chose a county seat because the infrastructure and background of the institutions in this city provide more opportunities for participation in extracurricular sports. Furthermore, the surrounding towns are within this large city’s catchment area, what is more, the cross-border regions are as well. Thus, we can find students from every social group in the various schools. However, the selective nature of the Hungarian school system from time to time, through its segregating actions, has, in certain school types, varying ratios of children of families with varied social status [33]. Therefore, in the course of choosing schools, we attempted to achieve a heterogeneous sample appropriate to be used as a population. Flowing from this, among the chosen schools could be found structure-shifting (six-grade secondary school) and primary, church-run, foundation- and state-funded, suburban and inner-city schools, and institutions with teachers of higher and lower socio-economic status students. The age group is an important criterion because of the success rate of completion and the higher level of parental involvement. School sports activities are more popular among primary school pupils, which justifies choosing this school type. Finally, in three of the primary schools visited, it was not possible to carry out the planned survey because the heads of the institutions refused to participate or did not reply to our requests. Thus, the sample of our research consisted of 7th and 8th-grade students from two primary schools in Debrecen and one six-grade secondary grammar school in another Hungarian city (N = 121). Data collection was carried out in the autumn of 2022.

We sought out the selected schools on three different occasions. First, we asked for the permission of the head of the institution and then contacted physical education teachers who helped us fill out the questionnaire. In the second round, in the schools which gave positive answers, we asked for permission from the parents of athlete-students in the classes to conduct the survey among their children. The questionnaire was only completed by students whose parents had indeed given permission and who were playing sports at some level, in an organized way. After acquiring permission, one week later, the students filled out the questionnaires in the presence of the researcher and the teacher during a P.E. class, which took approximately 30 min. In one school, the filling out of the surveys occurred once. 118 distributed questionnaires were filled out accurately and could be used for further analysis. For measuring consistency, we split the sample into two equal parts and compared the reliability of each, in the end coming up with similar results. Internal consistency of scales was measured by calculating the values of Chronbach’s α.

### 2.2 Participants

44.9% of the participants were girls, and 55.1% were boys. Furthermore, the average age of respondents is 13.02 ± 0.754.59.5% of the respondents learnt in the seventh and 40.5% in the eighth grades. Regarding parents' educational level, most of them have a tertiary educational degree. 97.5% of fathers and 95.7% of mothers are employed. 23.6% of respondents live in small towns, and 76.4% in large cities. The subjective financial situation of the students surveyed is as follows: 70.1% have everything they need and have savings, while 29.9% also have everything they need but cannot afford major expenses. 89.7% of students pursue sports in associations, 5.1% in schools and 5.1% in both. As the proportion of athletes in school sports is negligible, we were not able to compare the athletes pursuing sport in clubs and school. 44.9% of the respondents play competitive sports, 35.6% play sports as a hobby, and 19.5% play sports as a hobby but compete in amateur competitions. A higher rate of pupils play team sports (Table 1.).

**Table 1:** The sociocultural, demographic and sporting characteristics of the sample. Source: own research.

Variable	Value	Percentage	N
Gender	Male	44.9	118
	Female	55.1	
Age	12	27.4	117
	13	43.6	
	14	29.1	
Grade	7th	61	118
	8th	39	
Type of school	primary	79.3	116
	grammar	20.7	
Fathers' education	Primary	4.2	118
	Secondary	16.9	
	Tertiary	78.8	
Mothers' education	Primary	0.8	118
	Secondary	14.4	
	Tertiary	84.7	
Father’s employment	Employed	97.5	118
	Not employed	2.5	
Mother’s employment	Employed	95.7	117
	Not employed	4.3	
Type of settlement at age 14	Large city	76.4	110
	Small town	23.6	
Subjective financial status	everything have they need and have savings	70.1	117
	Everything have they need but cannot afford major expenses	29.9	
Level of sport activity	competitive	44.9	118
	hobby	35.6	
	hobby but compete in amateur competitions	19.5	
Form of sport activity	In association	89.7	117
	In school	5.1	
	In both	5.1	
Type of sport	Individual	45.2	115
	Group	54.8	

**Fig. 1:**
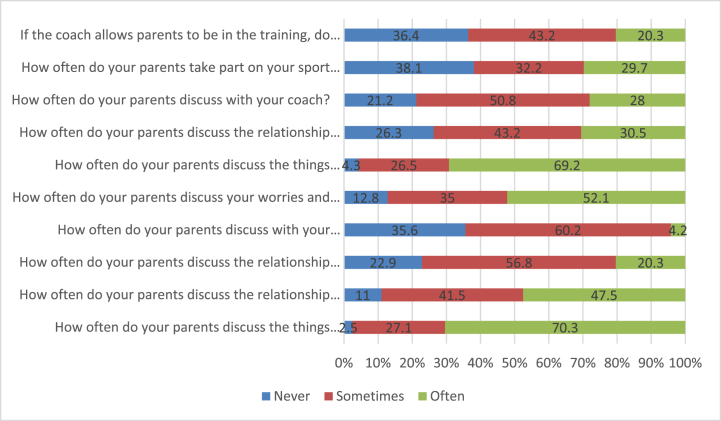
Frequency of different forms of parental involvement (N = 117–118). Source: own research.

From the answers to our questions on parental involvement, we can see that most parents never attend matches/tournaments and training sessions (38.1% and 36.4%, respectively). On the contrary, a high percentage of parents often ask their children about what happened at training (69.2%) and school (70.3%), often talk about their problems (52.1%) and about their friends (47.5%). The highest percentage of parents' communication with teachers and coaches is occasional (Fig. 1).

### 2.3 Measurements

The questionnaire contains 79 items, 2 open-ended (name of school and the sports) and 77 closed-ended questions. In our questionnaire, we investigated five main dimensions: 1. Forms of parental involvement, 2. Academic achievement, 3. Sporting achievement, 4. Health and health risk behavior (for this study these questions were not used), and 5. Sporting, sociocultural and demographic background. In the first dimension, we measured parental involvement by using the modified questions of Xia et al. [34], supplementing them with items on involvement in sports (5-5 items). The block of questions was reliable (Learning-related parental involvement: Cronbach α = 0.708, Parental involvement in sport: Cronbach α = 0.675). Using the items of the question block, cluster analysis was employed to form student groups, which were as follows: children of parents not involved (N = 33), children of parents involved in studies (N = 38), and children of parents involved in both studies and sports (N = 47). Parents involved in both studies and sport can be considered a group which interprets and supports their child’s interest in and talent for sport as a strength, i.e. they are characterised by strength-based parenting or even sport-strength-based parenting.

The extra attention and resources invested by parents in the education of athlete-students are evident (accompanying children to competitions and training sessions, adapting family life to the given sporting programme, providing equipment, transport, etc.). We can neither confirm nor deny the SBP approach in the case of the group of parents for whom we detected involvement only in studies, because we focused specifically on parental attitudes regarding sport-related effort and did not examine, for example, pupils who play music.

We measured academic and sporting performance broadly. We asked about the respondent’s best national and international sporting performance (2 items), whether they had ever received an award for academic or sporting achievement (4 items), and whether they were involved in any talent management programmes (2 items). We also asked how students rate their academic and sporting achievements and how their parents, peers, coaches, and teachers view their accomplishments (on a 5-point Likert scale, 8 items). Concerning plans, we asked, how likely the respondent was to play competitive or hobby sports in high school and after school (5 items). Commitment to sport was assessed using the Passion in Sport questionnaire [35] (11 items, Cronbach α = 0.806). Factor analysis was used to create two factors: harmonious passion (for example: ‘Sport is in balance with other activities in my life’; ‘This sport allows me to live different experiences’; ‘The sport I play is in harmony with everything else that is part of my life’ etc.) and obsessive passion (for example: ‘I am almost obsessed with sport’; ‘If I could, I would only play the sport of my choice’; ‘This sport is so exciting that sometimes I lose control, and I get lost in it’; ‘I have the feeling that the sport I play has control over me’ etc.) (maximum likelihood method, varimax rotation, KMO = 0.825, sig = 0.000, total variance explained: 44.686%) [32].

To explore the socio-cultural and demographic background, we examined the respondent’s gender, age, type of school chosen, educational level of parents, labour market status, type of settlement, and objective and subjective financial situations (19 items). We also asked about the type, form, and level of sport activity (4 items).

Due to reasons of volume, the logical arch of our study, and the preservation of its coherence, we did not examine the “health and risk behavior” in the current study; we plan to explore its connections to parental involvement and sport through the course of further analyses in another study.

Statistical analyses were performed using the SPSS 25 statistical software package. Descriptive statistical analyses, cluster analysis, factor analysis were used to create the student groups and the factors of HP and OP, while a Chi-square test, ANOVA and a Kruskal-Wallis test were used to examine the socio-cultural background of student groups pertaining to parental involvement and the differences in academic and sporting achievement between the student groups (H1 and H2). Linear regression was used to analyse the role of parental involvement in HP and OP (H3). Normality tests were performed using the Kolmogorov-Smirnov test. Interactions were measured and assessed with analysis of variance (ANOVA, F-test, *p*-values), and checked with an interaction plot.

## 3 Results

Examining students' social, demographic and sporting backgrounds, we found significant differences and large areas of effect between the student groups along the lines of sporting participation. 66.6% of children of parents involved in both studies and sports, and 45.5% of children of non-involved parents pursue competitive sports. At the same time, 60.5% of children of parents involved in both studies and sports play sports at a hobby level (Pearson chi square = 24.669, p = 0.000, Cramer’s V = 0.323) (Table 2). No significant differences between clusters were obtained for the other variables tested.

**Table 2:** Associations between level of sport participation and parental involvement (N = 118) Source: own research.

	Parents not involved	Parents involved in studies	Parents involved in both studies and sport
Competitive	45.5%	18.4%	66.6%
Hobby	39.4%	60.5%	35.6%
Hobby level, but I participate in amateur competitions	15.2%	21.1%	21.3%

*The underlined values indicate that the number of people in that cell of the table is much higher than would have been expected in the case of random ordering.

### 3.1 Differences in academic and sporting performance between groups of pupils

21.2% of the students' best international competition result was first place, 3.4% came in second place, 4.2% received third place, 5.9% placed below the top three, and 65.3% did not place at all. 37.9% of students' best national result was first place, 2.6% reached second place, 6% third place, 6.9% placed below the top three, and 46.6% did not place. 53.4% of the respondents have received an award or scholarship for their academic performance during the semester or at the end of the year, and 37.3% for their academic competition results. Less than half of the respondents (46.6%) had received an award for sports performance at school. 20.3% of students participate in a school-related talent programme, and 20.3% in a sports talent programme. We investigated the relationship between the different student clusters connected to the patterns of parental involvement and achievement. The proportion of children of parents who were involved in both studies and sports and had a first-place finish was over-represented for the best international competition result. However, no significant difference was found between clusters. A significant difference was found between the best national competition result and clusters with medium effect size (Pearson chi square = 15.633, p = 0.048, Cramer’s V = 0.260). Our results show that among children of parents involved both in studies and sports, almost 70% achieved some kind of placement in a national competition. In comparison, the proportion of children whose parents were involved in studies was over-represented among those who did not achieve a placement (68.4%) (Table 3).

**Table 3:** Correlations between best national competition result and parental involvement (N = 116) Source: own research.

	Parents not involved	Parents involved in studies	Parents involved in both studies and sport	Chi-square	Sig
1st place	43.8%	21.1%	47.8%	15.633	0.048
2nd place	0%	2.6%	4.3%		
3rd place	3.1%	2.6%	10.9%		
Point-scoring placement	9.4%	5.3%	6.5%		
No placement	43.8%	68.4%	30.4%		
	100%	100%	100%		

*The underlined values indicate that the number of people in that cell of the table is much higher than would have been expected in the case of random ordering.

When comparing student clusters and academic/athletic performance awards and scholarships, we found a significant difference in the academic performance and artistic/athletic performance awards with medium effect size (Pearson chi square = 7.301, p = 0.026, Cramer’s V = 0.249 and Pearson chi square = 7.985, p = 0.018, Cramer’s V = 0.260). The results reveal that a higher percentage of children of parents involved in studies received an award for their academic achievement (71.1%). In comparison, children of parents involved both in studies and sports received an award for their artistic/sports performance (59.6%). However, among the latter, 42.6% also received an award or scholarship for their outstanding academic performance. Among the children of non-involved parents, 48.5% received awards for both academic and artistic/sports performance. A low proportion of children of parents who were not involved in studies received an award for their sports performance (28.9%).

We examined the correlations between participation in talent management programmes and student groups based on parental involvement. We found a strong correlation between participation in a sports talent programme and parental involvement with medium effect size (Pearson chi square = 6.237, p = 0.044, Cramer’s V = 0.230).

Our data shows that children of parents involved in both studies and sports have the highest proportion of children participating in a sports talent programme (29.8%). In comparison, children of parents involved in studies only are underrepresented among programme participants (7.9%), and this proportion is 21.2% in the case of parents not involved at all.

Pupils were asked to rate their academic and sports performance according to their school grades and were also asked their views on how their parents, friends/teammates, teachers, and coaches rated their performance. We found significant differences between ratings and clusters in three cases with low size effect: self-ratings of academic and sports performance (F(2,115) = 5.2012, p = 0.007, η2 = 0.083 and F(2,115) = 3.461, p = 0.035, η2 = 0.057) and parental ratings of academic achievement (F(2,114) = 7.550, p = 0.001, η2 = 0.117).

Our data reveal that children of parents not involved in studies rated their school performance as the worst (M = 4.06, SD = 0.788). In contrast, children of parents involved in studies rated their school performance as the best (M = 4.57, SD = 0.603), and children of parents involved in both studies and sports scored M = 4.19 (SD = 0.537). Children of parents involved in studies also scored the highest mean scores in the parent rating (M = 4.63, SD = 0.657), children of parents involved in both studies and sports scored M = 4.28 (SD = 0.621), while children of parents not involved M = 3.94 (SD = 0.966). The children of parents involved In both studies and sports rated their sports performance best (M = 4.15, SD = 0.807), followed by children of parents not involved in either (M = 3.85, SD = 0.906) and finally by parents involved in studies only (M = 3.66, SD = 0.909).

### 3.2 Differences between clusters of pupils in plans and commitment to and passion for sport

In our questionnaire, we asked students how likely they were to pursue competitive or hobby sports during high school and after graduation. We found a significant relationship between competitive sports plans in high school and after graduation and parental involvement clusters with large effect size (Pearson chi square = 18.103, p = 0.020, Cramer’s V = 0.277 and Pearson chi square = 21.731, p = 0.001, Cramer’s V = 0.330).

From our data, we can see that the children of parents who are involved in studies and sports have the highest proportion of students who are very likely to participate in competitive sports during their high school years (57.4%) and after high school as well (55.3%).

Most children of parents involved in studies are more likely to pursue competitive sports during their high school years (28.9%). However, a proportion thinks that it is unlikely to play competitive sports after graduation (28.9%), and the majority of children whose parents are not involved in their studies (27.3%) also think so (Table 4).

**Table 4:** Correlations between future sports plans and parental involvement (N = 118). Source: own research.

		Parents not involved	Parents involved in studies	Parents involved in both studies and sport	Chi-square	Sig.
Likelihood of pursuing competitive sport during high school years	Not et al.	12.1%	13.2%	6,4%	18.103	0.02
	Rather no	24.2%	18.4%	10,6%		
	Rather ye	9.1%	28.9%	17,0%		
	Absolutely	45.5%	18.5%	57,4%		
	I don’t know	9.1%	21.1%	8,5%		
	N	100%	100%	100%		
Likelihood of pursuing competitive sport after graduation	Not et al.	27.3%	13.2%	2,1%	21.731	0.001
	Rather no	15.3%	28.9%	10,6%		
	Rather yes	15.2%	23.7%	17,0%		
	Absolutely	24.2%	15.8%	55,3%		
	I don’t know	18.2%	18.4%	14,9%		
	N	100%	100%	100%		

*The underlined values indicate that the number of people in that cell of the table is much higher than would have been expected in the case of random ordering.

Commitment to sport was measured using the Passion in Sport scale. No significant differences were found between student cluster groups along parental involvement regarding the items, except for the statement that controlling his desire to play sports is difficult. The results of the Kruskal-Wallis test show that children of parents who are not involved agree most with this statement (MR = 72.53), followed by children of parents who are involved in studies and sports (MR = 56.91), with a much lower ranking. In contrast, children of parents who are involved in studies agree the least (MR = 51.38) (Chi square = 7.627, p = 0.022). Two factors were developed: harmonious passion (HP) and obsessive passion (OP). We found a significant difference between clusters in OP, which shows low effect size (p = 0.048, F(2,113) = 3.122, η2 = 0.0.52). Children of parents involved in studies were found to be the least obsessed with sports (M = −0.17, SD = 0.94), followed by children of parents involved in both studies and sports (M = −0.11, SD = 1.02). Children of non-involved parents had the highest mean values (M = 0.37, SD = 0.95). Linear regression was used to examine the role of clusters of parental involvement, gender, parents’ educational level, objective financial status and type of settlement on the passion for sport, but no significant association was found for any of the variables. However, only involving two clusters of students (children of parents involved in studies and children of parents involved in both studies and sports) in the model, we found a significant negative correlation with OP (F(3,112) = 3,122, p = 0.048, R^2^ = 0.052, Tolerance: 0.668, VIF: 1.497). Belonging to both learning groups has a negative effect, reducing obsessive passion (βt = −0.259* [−1.019, −0.078], βt + s = −0.237* [−0.930, −0.030]) (Table 5.).

**Table 5:** Effects of parental involvement forms on OP of sport (linear regression coefficients) (N = 116). Source: own research.

Independent variables	B	Std. Error	Beta	t	Sig.	Lower Bound	Upper Bound
Parents involved in studies	−0.548	0.238	−0.259	−2.308	0.023	−1.019	−0.078
Parents involved in both studies and sport	−0.480	0.227	−0.237	−2.113	0.037	−0.930	−0.030

*Reference category: children of not involved parents.

## 3 Discussion

In our study, we investigated the associations of the different forms of parental involvement with the socio-cultural, demographic and athletic background, academic and sports performance, sport-related plans and passion for sport among 7th and 8th-grader students (N = 118) in three schools in a large Hungarian city. Among the dimensions of parental involvement, we focused primarily on parenting and were interested in the associations of strength-based parenting (SBP) with students' academic and sports performance. In our interpretation, following Waters [20], SBP is a positive approach of parenting, i.e. seeing the potential in the child, looking for his or her strengths, emphasising them and focusing on them. We investigated two types of parental involvement: involvement in studies and sports, based on which we distinguished three groups of students using cluster analysis [11]: those involved in studies (N = 38) [1], those involved in studies and sporting activities (N = 47, considered as the sample for SBP), and [2] children of parents who were not involved (N = 33). Interestingly, we could not detect a group of parents involved only in sporting activities. This may be due to the fact that, on the one hand, parents who primarily support their child’s sporting career also consider it important to simultaneously establish their child’s career. As part of this, they monitor and support academic careers and dual careers. Another explanation could be that, due to the non-cooperating schools, our sample is relatively homogeneous regarding the social background of the students, most of whom have a higher social status, as the vast majority of parents (78–84%) are graduates. Possibly, there were no students in the sample whose families occasionally or regularly had financial problems. Also, previous research has demonstrated that the socio-cultural background of the family determines the level of parental involvement: families with higher qualifications and better social backgrounds tend to have higher levels of parental involvement [2,6,12,22,36]. This also explains why we did not find significant differences between cluster groups in the socio-demographic background of the students. Thus, our first hypothesis was not confirmed. However, there was a trend towards an overrepresentation of children of non-graduate parents among children of non-involved parents and objectively worse financial situations, so it would be important to investigate this issue further in a larger and more socially heterogeneous sample.

In our second hypothesis, we assumed that children of parents involved in studies are more successful in their academic performance. Our hypothesis was confirmed, although we only found significant differences in three questions. In these questions, the children of parents involved in studies performed better. Subjectively, they rated their school performance as better, and the proportion of pupils who received some kind of reward for their academic achievement at the end of the semester/year was overrepresented. The strong parental attention is confirmed by the fact that pupils in this group also rated their parents' evaluation of their academic achievement higher. Previous research has demonstrated the positive impact of parental involvement on academic performance: children of parents who are more attentive to their children’s school career, relationships and personal development, daily problems, organise more joint activities, have stronger relationships with the school, especially with teachers, are more successful academically and less likely to drop out [2,6,7,12].

Our results also show that these parents see sport as a leisure activity developing their personality and health. Their children are mainly amateur athletes or occasionally participate in amateur competitions; few pursue competitive sports, and very few have achieved competitive results. This also shows that learning is the first priority, above all else, and most are unwilling to risk getting too involved in sporting activity for fear that it could overshadow or hinder their academic progress. This is also confirmed by the fact that belonging to this cluster explicitly protects against the obsession with sport so that these parents are more careful to ensure that sport (in whatever form and at whatever level) does not become a passion for their children.

In our third hypothesis, we hypothesised better sports performance and higher engagement of children of parents involved in sports. Their hypothesis was partially confirmed since although the children of parents who were also involved in sports were indeed more successful in the dimensions studied, the parents of this group of students were significantly involved not only in sports but also in studies. Not only do they monitor and support their children’s competitive careers, but they also do not neglect their school progress, as shown above. This is borne out by the fact that not only do these pupils score the highest average score in terms of obsessive passion for sport, but also they are over-represented among competitive athletes - the children of parents who are not involved. According to the results of the linear regression analysis in the latter case, parents may become insecure and underachieving in both areas if they do not pay attention to their children’s studies and sporting careers. They may find it difficult to balance studies and sports together, or even to set a ‘healthy’ limit when it comes to playing sports. On the contrary, parental involvement in their child’s studies, and even in sport and academics, has a markedly protective function against obsessive passion. As was previously stated, OP means that the individual loses control over the activity being performed, i.e. sport, which thus takes pre-eminence over all other activities, e.g. studying, and entails severe psychological costs [32]. This is similar to the scale of motivation regarding both academic and sports performance. Both extrinsic and intrinsic motivation can be useful in achieving the goals, even if intrinsic motivation is said to be more useful. However, when a balance is found, their combined effect can significantly contribute to the success of the student. Since both harmonious and obsessive passion arise from human motivation, we can conclude that finding and keeping the appropriate incentives behind our activities and aims can be the best way to be successful. Our results suggest that a higher degree of parental support and attention to both studies and sports controls the child’s passion for sport, not allowing it to be detrimental to other areas of the child’s life, including studies.

However, dual involvement has a positive effect mainly on sports performance: children of parents involved both in studies and sports have better results in both subjective and objective indicators. They rate their own sports performance as better. Also, among them, an over-representation can be seen concerning those who have received some kind of reward for their sporting achievements or are involved in a sports talent programme. We can see that these parents do not put all their eggs in one basket, forcing their children to be involved only and exclusively in sports, to be a top athlete, and consider an academic career important. Previous research has shown that a high level of parental involvement has a positive effect on sporting performance [25] and that high expectations can also be detrimental to performance [27]. Our results also confirm that parental attention to the children’s academic and sporting careers has a positive effect on sporting performance, which is consistent with the concept of SBP. The parents who are also involved in sport and studies have found the appropriate type of sport and sports club for their children, transporting them to and from there several times a week, and rearranging their family schedule to accommodate training and competitions, while also providing them with the equipment. Thus, they recognize and nurture their child’s strengths, valuing their child’s activities, i.e. this is parenting that focuses on sport as a strength. The importance of SBP has been highlighted by research based on positive psychology, and its relevance to child and parental well-being is prominent [17], as the competitive mindset often found in schools causes failure and anxiety for a significant proportion of students. However, if there is another area where the student is happy, enjoys the activity and is successful, this can alleviate the anxiety of academic underachievement. At the same time, even in the case of possible difficulties at school, it gives parents the confidence to see that their child has strengths and is persistent and diligent in training because it gives them hope that they will be able to excel in other areas in the long run.

The reason why there is a positive correlation between sports and academic performance when parents are also involved in sport can be explained in three ways. The first, as mentioned above, is the importance of dual career building for parents. Such attention to academic history has a positive effect on academic achievement, as shown by Epstein and others [1,2,6,7,12,22], and involvement in sport likewise has an impact on sports performance [17]. A second explanation may be the mediating role of higher well-being indicators associated with SBP, such as mental toughness, strength-based coping strategies and perseverance, which positively correlate with higher academic performance [16,17]. The third explanation lies in the positive, personality-developing effects of the sporting activity, such as teaching individuals to respect hard work and to be persistent. It also improves cognitive skills and enhances self-confidence, competitiveness, and responsibility. These are also positively associated with academic achievement [[8], [9], [10]].

A limitation of our study can be found in the small and non-representative sample, which mainly includes children from families with higher socio-economic backgrounds. The need to increase the number of items in the sample is confirmed by the low effect size in ANOVAs. In our further research, we aim to expand the sample to include students of lower social status. A further limitation is the lack of the examination of all six dimensions of parental involvement based on the Epstein model. Furthermore, it would be important to include mediating factors to assess the direct and indirect effects of parental involvement. Unfortunately, due to the specific situation of the Hungarian school sports system and the fact that this issue is also missing from international studies, there is no possibility to explore the relationship between parental involvement in classical school sports and academic performance.

Future studies should aim to set up and test a multivariate model in which the factors influencing SBP (socio-cultural, ethnic, demographic background, motivation, etc.) would be investigated, and its direct and indirect predictive effects on academic and sports performance, by including different indicators of well-being, such as mental toughness, commitment and perseverance, in a larger and more heterogeneous sample in terms of social background.

Future studies should also eliminate the above limitations to enhance understanding of the relationships between parental involvement, academic performance, and sports. Based on these, it would be possible to see whether it is true that among low social status students the extent of parental involvement is truly lower or not, and, in those disadvantaged families where it is not, whether or not there is a compensating role in students' academic and sport performance, whether parental involvement makes them resilient or not in the face of worse performance or even drop-out. The multi-dimensional examination of parental involvement would contribute to uncovering its diversity, its various forms, and thus we would perhaps be able to discover more differences in effectiveness, and regarding the background of the parent types. Including intermediary factors in the examination of ties and in the discovery of the latter helps us know whether there is a direct relationship between parental involvement and effectiveness, or through another of the students’ indicators (as well) (e.g.- well-being, confidence, mental toughness, commitment, etc.).

## 5 Conclusions

In our study, we investigated the socio-cultural background of parental involvement in studies and sports in primary schools in a large Hungarian city and its relationship with academic and sports performance. One of our study’s most important novum is, while earlier, the effect of parental involvement on academic achievement [1,2,6,7,12,22], and the parental presence in sports and sport performance was measured by others [17], we combined the above types of involvement, and, in both dimensions, uncovered the roles of these two types of parental involvement. The most important results of our research were that parental involvement has a positive impact on academic and sports performance, and it is a negative predictor of OP for sport. Compared to previous literature, one of the novel findings of our research is that better sport performance is more likely for students whose parents are both attentive to and supportive of their children’s academic and sporting careers, while parents involved in studies only are primarily concerned with their child’s academic career, perhaps that is why they tend to keep them away from competitive sport. An important finding is that children of non-involved parents also have a high level of sporting performance, which is associated with a higher level of obsession with sport. At the same time, parental involvement in sports and studies also acts as a protective factor against this obsession. Therefore, it is proposed that sports organizations should implement intervention programmes to involve parents in the (sporting) life of the organisation and of the children. These would provide regular information regarding the child’s performance and would allow parents to ask questions of and express opinions to, address difficulties, and dilemmas with sports administrators and coaches on the compatibility of sports and studies. In addition, they can organise joint activities to get to know each other (parents, actors in sports and children). Educational programmes should also be implemented to help athletes and their parents plan and pursue dual careers, and to support children in planning and achieving their sporting ambitions and goals. Similar programmes are needed for sporting children in educational institutions to help them and their parents to pursue a potential career in sport alongside excellence in academic achievement so that no sporting talent is lost. At the same time, it is worthwhile for sports organizations to cooperate with an athlete’s teachers, that the teachers would not look on doing sports as something that would draw students away, but rather as a resource, which develops multiple skills and behavioral norms. It could also be a positive example in the student community.

It was an unexpected result in our study that if the parent does not pay enough attention to their child’s academic and sports career, then his/her child will be at greater risk of becoming a sports fanatic. It would be critical to examine what lies behind this, and what defensive measure or factors could prevent this from occurring. The present study confirms the fact that if a parent is present and follows attentively his/her child’s academic and sporting careers, then that contributes to finding the correct balance between sports and studies, but, it must not be forgotten that the coach must also be a partner in this process. It is paramount that he consults with the parents about the child’s development and performance in the sport, but, equally important, is that he be informed by parents and teachers about how the sport can be harmonized with the student’s academics and the other areas of his/her life in the interest of finding the right balance, and in the interest of putting sport in the right place in the lives of both the child and the parents. That is why such parent–coach– teacher meetings, perhaps even conducted with the help of a professional, would be necessary, where they could pose their questions and problems, search for answers and solutions in order to find that academics-sports balance.

As another research direction in regards to this, we could examine what sort of connections appear between the various forms of parental involvement which appear in the Epstein model, specifically participation in the lives of the school and the sports club, as well as the forms, frequency, etc. of communication with teachers, coaches, and leaders, and the academic and athletic performance of athletic students. Also important would be the examination of what sort of factors lie behind the forms of parental involvement, that is, what influences certain parents to, in what form and with what regularity, participate in the life of educational and sport institutions.

Although our study’s uniqueness is found in how the many forms and roles of parental involvement were equally examined regarding academic and athletic achievement, as regards the socio-economic indicators and the indicators of the number of items in the sample, due to their homogenization, the results cannot be generalized. Thus, the expansion of the sample, as well as including the moderator factors would be important for continuing the research. In our study, however, it was clearly confirmed that parental involvement in academics and athletics plays a positive role in both the scrutinized areas of achievement, and, based on this, such prevention programs can be developed in which strengthened parental involvement can help successfully realize a sporting student’s dual career, along with finding his/her health balance between studies and a career in sports.

## Data availability statement

The study was conducted in accordance with the Declaration of Helsinki, and approved by the Institutional Review Board (or Ethics Committee) of the School Ethics Committee of Doctoral Program on Educational Sciences at the University of Debrecen (protocol code 1/2022 and date of approval: 09 March 2022). The research was conducted ethically, the results are reported honestly, the submitted work is original and not (self-)plagiarized, and authorship reflects the individuals’ contributions. Data associated with the study has not been deposited into a publicly available repository. The data that has been used is confidential.

## CRediT authorship contribution statement

**Klára Kovács:** Writing - review & editing, Writing - original draft, Visualization, Methodology, Investigation, Formal analysis, Conceptualization. **Ádám József Oláh:** Writing - original draft, Resources, Investigation, Data curation, Conceptualization. **Gabriella Pusztai:** Writing - review & editing, Writing - original draft, Supervision, Investigation, Funding acquisition, Conceptualization.

## Declaration of competing interest

The authors declare that they have no known competing financial interests or personal relationships that could have appeared to influence the work reported in this paper.
